# Drupe Characters, Fatty Acids, Polyphenolic and Aromatic Profile of Olive Oil Obtained from “Oliva Bianca”, Minor Autochthonous Cultivar of Campania

**DOI:** 10.3390/plants10061119

**Published:** 2021-05-31

**Authors:** Claudio Di Vaio, Giulia Graziani, Anna Gaspari, Lucia De Luca, Alessandra Aiello, Aurora Cirillo, Antonio Bruno, Raffaele Romano, Alberto Ritieni

**Affiliations:** 1 Department of Agricultural Sciences, University of Naples Federico II, Via Università 100, 80055 Portici, Italy; claudio.divaio@unina.it (C.D.V.); lucia.deluca@unina.it (L.D.L.); alessandra.aiello@unina.it (A.A.); aurora.cirillo@unina.it (A.C.); antonio.bruno3@unina.it (A.B.); 2 Department of Pharmacy, University of Naples Federico II, Via Domenico Montesano 49, 80131 Naples, Italy; annagaspari@virgilio.it (A.G.); alberto.ritieni@unina.it (A.R.); 3 Unesco Chair for Health Education and Sustainable Development, 80131 Naples, Italy

**Keywords:** germplasm, characterization olive oil, phenolic profile, volatile compounds, cultivar

## Abstract

Campania, due to its pedo-climatic conditions and to its large varietal heritage, is able to produce oils with high typicity, each different from the other. In this study a “minor” autochthonous cultivar of Campania “Oliva Bianca” was analyzed. In autumn 2020, on drupes from trees belonging to the Campania germplasm collection a varietal characterization through physical, chemical and chromatic parameters at the harvest was carried out. Phenolic compounds profile, fatty acids composition and volatile organic compounds have been investigated in the resulting oil. Quality indices, organoleptic and sensory qualities (panel test) were also determined on the oil. Drupe weight was 4.31 g, flesh/pit ratio was 3.68 and the accumulation of oil content at harvest in drupes was 18.63% FW. The drupes showed high anthocyanins content equal to 116.10 mg/kg. In the oil studied, the secoiridoids represented the 82.25% of total phenolic compounds, the concentration of oleic acid was 74.82% and the most present volatile compound was trans-2-hexenal (72.30%). High secoiridoid derivatives concentrations such as oleuropein (85.93 mg/kg) and ligstroside (122.43 mg/kg) aglycones were showed. This study showed a good content of qualitative and quantitative parameters of “Oliva Bianca” oil and drupe, that can have important beneficial effects on human health.

## 1. Introduction

The olive (*Olea europaea* L. subsp. *europaea*), which originated in the Eastern Mediterranean area, has been cultivated throughout the Mediterranean basin since ancient times for its fruit and oil. It is believed to have been domesticated as far back as 3500–3700 BC and references to the use and trade of olive oil date back to 2000–3000 BC [1]. In Italy, each region has its own local cultivars, and many seedling trees grow spontaneously and the high genotypic diversity of olive varieties could be explained by human selection in response to local environmental and agronomic conditions [2,3]. The larger olive producing Italian regions are Apulia, Calabria, Campania and Sicily. The use of the species, both as table and oil cultivars, is well-documented in these regions, with many archaeological and written relics dating back to ancient times, attested also by the presence of monumental trees [4]. It is known that olive cultivar differentiation based on morphological descriptions is not particularly reliable, as it can be influenced by environmental conditions and requires skilled staff [5]. Furthermore, the presence of both native and foreign olive cultivars with ambiguous naming together with the interchange of plant material over the centuries, make it difficult to ensure cultivar identification and to fully understand the pattern of geographic distribution [6].

Varieties represent the most important strategic element to boost the commercial value of Italian extra virgin olive oil. Thanks to its varied pedo-climatic conditions, Campania has a very rich gene pool of olive cultivars, which have adapted in the course of millennia to different agro-ecosystems. This important source of biodiversity must be preserved and exploited to avoid the risk of genetic erosion [7].

Olives and olive derived are an important valuable source of natural phenolic antioxidants [8]. In fact, an increasing number of epidemiologic and experimental studies report that the olive oil may have a role in the prevention of coronary heart disease [9], cognitive impairment, e.g., Alzheimer’s disease [10], protective effects against of the cancer of the colon, breast and ovary [11], diabetes accompanied by hypertriglyceridemia and inflammatory and autoimmune diseases, such as rheumatoid arthritis [12].

In Campania many of the traditional cultivars are exclusively used for oil production; only few cultivars, such as ‘Ortice’, ‘Ortolana’ and ‘Caiazzana’ are suitable for use both as table olives and oil production. Campanian olive oils vary considerably and possess typical and distinctive sensory properties [4,13].

In this study we examined a minor autochthonous cultivar of Campania called “Oliva Bianca”, studying the morphological of drupe and the oil chemical characteristics. The “Oliva Bianca” is a cultivar from Salerno province within the territory of the “Cilento e Vallo di Diano” National Park.

## 2. Results

### 2.1. Physical and Chemical Characters of Drupes

A previous study polyannual was conducted from 2003 to 2009 on the germplasm of Campania involving 20 autochthonous olive cultivars from Campania including the “Oliva Bianca” cultivar. In this study the cultivar “Oliva Bianca” has shown a cumulative production efficiency greater than the other cultivars analyzed (0.67 kg/cm^2^), due to a high cumulative production per plant (43.47 kg per plant) and a reduced trunk cross-section area (130 cm^2^) [7].

In terms of fruit size, the “Oliva Bianca” shows a high weight drupe with 4.31 ± 0.90 g, formed for 78% by flesh, flesh weight equal to 3.36 ± 0.80 (g), pit weight of 0.95 ± 0.23 (g) and a flesh/pit ratio equal to 3.68 ± 1.00 (Table 1).

The “Oliva Bianca” drupes have a spherical fruit (L/I = 1.25–1.45) and rounded base with a polar diameter mean of 21.41 ± 1.75 (mm) and transversal diameter mean of 17.53 ± 1.29 (mm). Regarding pulp firmness, this cultivar generally showed high resistance to penetration, in fact, the value for this parameter was 460.45 ± 34.98 (kg/cm^2^) at veraison.

The “Oliva Bianca” is characterized by both a late flowering (25–28 May) and late veraison (10–30 Nov).

The ripening of the fruits at harvest was determined by the Jaén-index, according to which the optimal harvest time is when the value lies between 2 and 3, in our samples of the cultivar “Olive Bianca” showing (PI) value of 2.41 ± 0.35. The peel color was also determined by chromatic parameters (L* a* b* and IC), L* showed value of 46.45 ± 12.2, a* (red direction) of 6.34 ± 9.62 and major value b* (yellow direction) of 16.9 ± 15.56, these data show weak skin veraison, index color showed the value of -32.86 ± 1072.93. 

The chemical profile of the fruits showed a high anthocyanins content equal to 116.1 ± 0.4 (mg/kg), carotenoids equal to 2.1 ± 0.01(mg/kg) and total chlorophyll of 5.7 ± 0.01 (chlorophyll a: 3.6 ± 0.03 and chlorophyll b: 2.1 ± 0.01).The accumulation of oil content at harvest in Oliva Bianca drupes was 18.63 ± 0.28 (%) in agreement with previous studies on the same cultivar which showed high values of 21.25% (%) [7], water content was 62.97 ± 0.17 in olive fruits.

### 2.2. Quality Indices of Olive Oil and Phenolic Compounds

In Table 2 quality indices of “Oliva Bianca” oil were shown. Free acidity was equal to 0.51% oleic acid, number of peroxides was 4.92 meq O_2_/ kg oil, K_232_ equal to 1.64, delta K less than 0.01 and K_270_ equal to 0.09. 

The individual phenolic compounds were identified and quantified by UHPLC coupled with high resolution mass spectrometry (HRMS) by means of comparison with available standards. As some standards were not available (the case of secoiridoids), quantitation was carried out employing calibration curves of available standards belonging to the same chemical group and with similar response (oleuropein) while for the identification MS/MS experiments had to be used. UHPLC-HRMS analysis allowed the separation and identification of 17 phenolic compounds, which may be grouped in the phenolic alcohols, phenolic acids, secoiridoids and flavonoids. In Table 3 the qualiquantitative results were shown.

In the oil studied, the secoiridoids represented the 82.25% of total phenolic compounds, however, hydroxytyrosol and tyrosol accounted together for 4.37% of total polyphenolic content. The dialdehydic form of elenoic acid linked to the tyrosol (p-HPEA-EDA) known as oleocanthal and responsible of anti-inflammatory activity was the main secoiridoid with a content of 255.20 mg/kg together with p-HPEA-EA (ligstroside aglycone) and DHPEA-EA (oleuropein aglycone) that were found at level of 122.43 and 85.93 mg/kg, respectively. DHPEA-EDA (oleacein), 3,4-DHPEA-AC (hydroxytyrosol acetate) and hydroxy oleuropein aglycon were detected in low amounts with concentrations of 33.43, 35.45 and 2.35 mg/kg, respectively.

The concentration of elenolic acid was 66.70 mg/kg, tyrosol and hydroxytyrosol, deriving from the hydrolysis of ligstroside and oleuropein respectively, were detected in the investigated oil in low concentration equal to 16.45 and 11.98 mg/kg, respectively. A good content of the flavonoids (luteolin and luteolin rutinoside) was observed with concentration of 17.37 mg/kg.

### 2.3. Fatty Acid Profile 

The fatty acid (FA) profile in Table 4 was shown. Palmitic (C16:0), palmitoleic (C16:1), heptadecanoic (C17:0), stearic (C18:0), oleic (C18:1), linoleic (C18:2), linolenic (C18:3), arachidic (C20:0), eicosadienoic acid (C20:2) and behenic acid (C22:0) were found. The most present FAs were oleic acid (74.82%), palmitic acid (12.87%) and linoleic acid (7.88%), while linolenic acid was present at 0.74%.

The concentrations of total saturated fatty acids (SFAs) and polyunsaturated fatty acids (PUFAs) were 15.70% and 8.63%, respectively. The MUFA/PUFA ratio was 8.77, while MUFA/SFA ratio was 4.82.

### 2.4. Aromatic Profile 

In Table 5 the results of qualitative analysis of VOCs were shown. 

The most present compounds were trans-2-hexenal that represented 72.30% of total VOCs, trans-2-hexen-1-ol (7.75%), hexanol (3.34%), ethanol (2.93%), ethyl acetate (3.60%), 1-penten-3-one (2.52%), ethyl-1,5-octadiene (1.83%) and β-ocimene. Compounds that were present below 1% were (t, t) 2,4-hexadienal, 4,8-dimethyl-1,7-nonadiene, cis-3-hexenyl acetate, nonanal, 1,7-octadien-3-one, 2 methyl-6-methylene and α-cubebene, which have also been found in other oils deriving from different cultivars and that influence the flavor of olive oil [14,15,16,17,18,19,20].

Regarding the panel test analysis, the oil must be considered as an extra virgin olive oil (EVOO) since the median of the fruitiness of 5.2 (medium fruity), 3.3 of the bitterness (medium bitterness) and 4.7 of the pungent of 4.7 (medium pungent) and that the median of the defects is 0, in fact according to the Regulation (EC Reg. 1604/2019) an oil in order to be defined extra virgin must have a median of defects equal to 0 and a fruitiness greater than 0.

## 3. Discussion

### 3.1. Physical and Chemical Characters of Drupes

“Oliva Bianca” is a variety very appreciated for its productivity, yield and some agronomic characteristics. In the present study, in agreement with the UPOV method, the “Oliva Bianca” drupes are considered to be of medium size (from 2 to 4 g), of oval-shaped and with rounded apex. Due to its high weight and the pulp/pit ratio (very close to 4), although this cultivar is mainly used for oil production, the drupes are also suitable and potentially marketable as table olives. When used for this purpose, olives are harvested after complete maturation, which gives to them a naturally black color due to anthocyanin accumulation [21]. The high content of anthocyanins (Table 1) are of great interest for human health because of their antioxidant activity and properties with respect to cancer prevention, inflammatory disorders, and cardiovascular diseases [22,23].

“Oliva bianca” cultivar is so called because at the veraison the fruits get a very pale color (white) before the appearance of the violet color. This cultivar is considered excellent all for its quality of the oil and it is characterized by a high oil content, in agreement with a previous study carried out on the same cultivar, which showed an oil content of fruits of 20–21% (FW) [7].

### 3.2. Quality Indices of Olive Oil and Phenolic Compounds

Our results were under the limits reported by Regulation EEC 1604/2019 of the European Union Commission, showing that the “Oliva Bianca oil” obtained has all the characteristics to be considered EVOO (Table 2).

EVOO is the highest quality of olive oil; edible olive oils are graded into EVOO with acidity up to 0.8%, calculated as oleic acid. Furthermore, an EVOO must have the following parameters: peroxide value PV (maximum value: 20 meq of O_2_/kg oil), K_232_ and K_270_ absorbance at specific wavelengths (maximum values: 2.5 and 0.22, respectively) and acidity up to 0.8%.

As reported in previous studies [24,25] we found that the virgin olive oil under study contains low amounts of phenyl alcohols and phenyl acids and high concentrations of secoiridoid derivatives such as oleuropein and ligstroside aglycons, which originate from the oleureuropein, dimethyloleuropein and ligstroside glycosides found in olives (Table 3). The secoiridoid are the polar phenolic compounds of VOO with the highest antioxidant properties [26]. The flavonoid content found in the analyzed oil was also higher than that reported for oils obtained from the most common varieties of olives such as Frantoio, Leccino, Itrana and Coratina [27]. Our findings concerning “Oliva Bianca” oil, compared to literature showed some quantitative differences principally with regard to the main secoiridoids.

Interestingly, the oleacein level in the “Oliva Bianca” oil was lower than that reported for the oils produced by common olive varieties in the world such as Coratina, Itrana and Leccino but at the same time greater than that reported for the oil obtained from the cultivar Frantoio [27]. Finally, it is worth noting that oleocanthal (p.HPEA-EDA), a secoiridoid derivative with very promising pharmacological properties showed amounts that are among the highest reported in literature for extra VOO [28,29]. The oil investigated was in line with the health claim approved for the European Food Safety Authority (EFSA) according to which the expected beneficial effects occur with a daily intake of 20 g of oil with at least 5 mg of tyrosol, hydroxytyrosol and its derivatives. (EC Reg. 432/2012). Content of the flavonoids was higher than that found in the oil extracted from olives of three autochthonous Sardinian cultivars [30].

### 3.3. Fatty Acid Profile

The concentration of palmitic (12.87%), stearic (2.32%) and linolenic acid (0.74%) (Table 4) was similar to [31] that showing a concentration of 13.59%, 2.41% and 0.72% respectively, while the content of linoleic acid (7.88%) was lower compared to [31] that showing a concentration of 10.90% and the content of oleic acid (74.82%) was higher than [31] that reporting a concentration of 67.20% in olive oil derived from olive of Mediterranean harvest region. Furthermore, the oleic acid and palmitic acid contents were similar to [7] that showing in olive oil derived from cultivar “Ortice” a concentration range from 56.70% to 65.29% for oleic acid and a range between 13.93% and 16.33% for palmitic acid depending on environmental factors. The concentration of linoleic acid, arachidic acid (0.37%) and total PUFA (8.63%) was in accordance with [32] that showed a concentration ranging from 3.78% to 9.91%, from 0.14% to 0.46% and from 4.33% to 10.53% respectively in olive oil obtained by different olive varieties, while the content of oleic acid (74.82%), total MUFA (75.68%) and stearic acid (2.32%) were lower than [32] that showed a concentration that ranged from 78.20% to 81.70%, from 78.54% to 85.20% and from 2.37 to 3.01%, respectively in olive oil obtained by different olive varieties. The concentration of total SFA (15.70%), palmitic acid (12.87%), palmitoleic acid (0,85%) and linolenic acid (0.74%) was higher than [32] that showing a range from 10.23% to 13.45%, from 8.02% to 9.82%, from 0.31 to 0.62% and from 0.48% to 0.66%, respectively. 

The difference in FAs composition could be derived from pedoclimatic conditions, variety, altitude and climatic conditions that influence drupe ripening and composition [33]. Yun and Surh (2012) [34] verified that FA composition has a great influence on oil oxidative stability and the MUFA/PUFA ratio represents a key factor. 

The MUFA/PUFA ratio was similar to data of [32,35] that reported a ratio of MUFA/PUFA that ranged from 3.95 to 14.1 and from 8.27 to 17.10, respectively. Furthermore, the ratio MUFA/SFA was similar to Pinelli et al. (2003) [35] a range from 4.3 to 6.8 of MUFA/SFA in olive oil obtained from autochthonous cultivars growing in a specific Tuscan area.

Finally, FA composition influences the sensorial properties of oil and it is also an important marker for biodiversity and quality of product [36].

### 3.4. Aromatic Profile

The aroma of olive oil is attributed to aldehydes, alcohols, esters, hydrocarbons, ketones and furans [37]. As for the “Oliva Bianca” in our analysis (Table 5), also Caponio et al. (2014) [38] showed a percentage trans-2-hexenal that ranged between 76.11% and 78.87% in oil of Coratina cultivar depending on decanter processing parameters. Furthermore, Cevik et al. (2016) [39] showed a concentration of trans-2-hexenal in the range of 48.45-78.64% for oil from spotted fruits, 32.11–74.52% for oil from purple fruits and 26.73-65.03% for oil from black fruit. This aldehyde is derived from the metabolism of α-linolenic acid through the lipoxygenase (LOX) pathway, which influences the taste of green, pungent, bitter fruity, apple, almond and cut grass [40,41]. Trans-2-hexen-1-ol has green and pungent notes and an odor described as banana-green deriving from the reduction trans-2-hexenal by alcohol dehydrogenase (ADH) [18,20]. Hexanol influences the flavor of green fruity, floral and alcoholic [15,18], it originated from hexanal through ADH activity [41]. Ethanol was also detected deriving from anaerobic fermentation and influencing the flavor of apple-like and alcoholic, floral [17,42]. 

Ethyl acetate resulting from anaerobic fermentation and influencing the flavor described as vinegar, wine and sweet was well correlated to the winey-vinegar defect [17,19]. 

Among ketones, 1-penten-3-one was identified and it could be derived from a contact with metal surfaces and 13-alcoxy radical [17] the flavor of metallic, sweet, green and spicy [17,42]. Although not contributing to bitter taste, the occurrence 1-penten-3-one is positively correlated with bitter and pungent taste [41]. Furthermore, [43] showed that the concentration of 1-penten-3-one is influenced by cultivar and ripening period. Ethyl-1,5-octadiene gives a smell of green and it is also detected in slightly rancid and moldy oils [15,44]. β-ocimene in oils derived from fruits infested with *Bactrocera oleae* [16,20] but it was also detected in filtered and unfiltered oil of cultivar Ravece [45] and its concentration was influenced by pedoclimatic conditions [46]. Methyl benzoate affects a pungent and greasy taste [18,19,44].

## 4. Materials and Methods

### 4.1. Plant Material

The study was conducted in 2020 on olive trees belonging to the germplasm collection of Campania grown at the ‘Improsta’ experimental farm (Salerno—Southern Italy), situated about 100 m above sea level (40°37′01′′ N, 15°03′23′′ E). The studied olive genotype was a Campania minor cultivar called “Oliva Bianca”. Cultural practices at the orchard are usual in the production area, and no irrigation is supplied. In October from 4 trees in full production were picked by hand 120 kg of healthy olives and carefully stored in ventilated boxes and processed within 4 h.

### 4.2. Physical and Chemical Characters of Drupes

On a sample of 100 olives, drupe and pit weight with an electronic digital balance (Precisa Instruments AG, model XB220A, Dietikon, Switzerland), equatorial and polar diameters using a digital vernier caliper (Mitutoyo, Kawasaki, Japan ± 0.01) were determined. The same sample was used to determine pulp firmness (kg/cm^2^) by a penetrometer with a point of 1 mm in diameter. The ripening index (0–7) for olives was determined according to the method proposed by the “Estación de Olivicultura of Spain” [47]. This method is based on changes in skin and pulp colors. Samples of 100 fruits were taken randomly and classified into eight groups or categories: green intense (group A = 0), yellow or yellowish green (group B = 1), green with reddish spots (group C = 2), reddish or light violet (group D = 3), black with white pulp (group E = 4), black with <50% purple flesh (group F = 5), black with ≥50% purple flesh (group G = 6) and black with 100% purple flesh (group H = 7). Pigmentation index was calculated as ∑ (Aini)/N, where A is the group number, n is the fruit number in that group and N is the number of samples. Drupe water content was determined by difference between fresh weight and dry weight obtained by dehydration on a sample of one hundred fruits (in an oven at 105 °C until constant weight). 

The color of fruits was determined, also, with a colorimeter (Minolta, model CR-400, Tokyo, Japan) that was capable of quantifying colors according to international standards and expressed in define color spaces. The instrument was calibrated with “white” managed by the light source on a white tile, before each measurement. The L* a* b* (CIELAB) color space is the most common for measuring the color of an object or materials of different origins and it is widely used in all sectors. In this color space, L * indicates brightness, while a* and b* the chromaticity coordinates: +a * is the direction of red, −a * is the direction of green, +b* is the direction of yellow and −b * is the direction of blue [48] subsequently, the colorimetric index (IC) was calculated using the formula: IC = (1000*a)/(b*L).

### 4.3. Olive Oil Extraction

Oil extraction was performed using a micro-mill (Oliomio, type Mini 50, Florence Italy), kneading the fresh olive paste at 28 °C for 30 min. Subsequently, the oil was obtained using a Soxhlet extractor (Semi-automatic Solvent Extractor SER 148/3, VELP Scientifica Srl, Provincia di Monza e della Brianza, Italy) with n-hexane as a solvent. After 4 h of extraction, consisting in two discontinuous cycles at 130 °C, the sample was weighed for oil yield determination. The oil was filtered and stored at 15–18 °C in absence of light prior to analysis.

### 4.4. Quality Indices of Olive Oil

The olive oil extract was analyzed for quality indices: acidity (% oleic acid per 100 g oil), peroxide (meq O_2_/kg oil), spectrophotometric indices (K232, K270 and ΔK) and sensory profile by panel test according to the EC Reg. 2568/1991 and later amendments and International Olive Council (IOC) standard methods. The parameters K232 and K270 are the oil absorbance at 232 and 270 nm, respectively, and ΔK was calculated from the absorbances detected at 262, 268 and 274 nm using a Shimadzu UV-1601 spectrophotometer (Shimadzu, Kyoto, Japan).

Sensory analysis was carried out by eight assessors who were fully trained in the evaluation of VOO according to the official methods of the IOC (1996) and EC Reg. 1604/2019. Panel test was performed in the Department of Agriculture, University of Naples Federico II (Italy), using the evaluation form regulated by EC Reg. 640/2008 and reported in Table 1.

### 4.5. Fatty Acid Profile

The determination of fatty acid profile was performed by analyzing the fatty acid methyl esters (FAMEs) obtained after trans-esterification (EC Reg. 1604/2019; [49]). A 1% solution of oil extracted in hexane was prepared, and 300 µL of a 2 N KOH solution in methanol was added to 1 mL of this solution. After shaking with a vortex, phase separation occurred. A total of 1 µL of the upper layer, containing the FAMEs was injected into an Agilent Technologies 6890N gas chromatograph equipped with a capillary column (100 m × 0.25 mm inner diameter, film thickness of 0.20 μm) with a poly stationary phase (90% biscyanopropyl/10% cyanopropylphenyl siloxane) (Supelco, Bellefonte, USA), a hydrogen flame ionization detector (FID) and a programmed temperature vaporizer (PTV). The carrier gas used was helium at a flow rate of 2 mL/min. The oven temperature program was as follows: 140 °C for 5 min, 4 °C min^−1^ ramp to 175 °C for 20 min and then 3 °C min^−1^ ramp to 240 °C for 20 min. The PTV program was set such as follow: 60 °C for 0.1 min, ramp to 500 °C min^−1^ to 260 °C for 5 min. The split ratio was 15:1, and the flame detector temperature was set at 260 °C, while the injector temperature was set to 120 °C.

The identification of the compounds was carried out by comparison with a mixture of standards: FAME C4-C24, (Sigma-Aldrich fatty acid methyl esters 37 components). The results were expressed as % *w*/*w*.

### 4.6. Phenolic Compounds

#### 4.6.1. Chemicals

All the standards used were for the analysis were from Sigma Aldrich St. Louis, MO, USA, while hydroxytyrosol was purchased from Indofine (Hillsborough, NJ, USA), secologanoside from ChemFaces Biochemical Co., Ltd. (Wuhan, China) and oleuropein form Extrasynthese (Genay, France). Acetonitrile and water (LC-MS grade) were acquired from Carlo Erba reagents (Milan, Italy), whereas acetic acid (98–100%) was purchased from Fluka (Milan, Italy).

#### 4.6.2. Extraction of Phenolics from the Olive Oil

The extraction of phenolic compounds was carried out in accordance with that reported by Dini et al. 2020 [24]. Briefly, 25 g of oil were dissolved in 25 mL hexane, then three extractions with 15 mL of a mixture of methanol:water (3:2 *v*/*v*) to extract polar compounds. Subsequently, the three supernatants were combined and counter-extracted with 25 mL of hexane. The solvent was dried in a rotary evaporator (Buchi, Switzerland) at 40 °C. Finally, the residue was resuspended in 1 mL of methanol, filtered through 0.2 mm nylon filer and kept at 18 °C until analysis.

#### 4.6.3. Ultra High-Performance Liquid Chromatograph-Mass Spectrometry Analysis

To identify and quantify the phenols was used an Ultra-High-Performance Liquid Chromatography (UHPLC, Thermo Fisher Scientific, Waltham, MA, USA) equipped with a degassing system (Dionex Ultimate 3000), a quaternary UHPLC pump working at 1250 bar, an autosampler device, and a thermostated column compartment (T = 30 °C). The column was an Accucore a Q 2.6 µm (100 mm × 2.1 mm) (Thermo Scientific, Waltham, MA, USA) and the injection volume was 5 µL. About the mobile phases they were phase A (H_2_O 0.1% of acetic acid) and phase B (Acetonitrile). The flow used to elute the phenols was 0.4 mL/min while gradient was as follows: 0–5 min 5% phase B, 6–25 min 40% phase B, 25.1–27 min 100% phase B, 27.1–35 min 5% phase B and 35.1–45 min 0% phase B. To identify the phenolic compounds a comparison was made between retention time and mass spectra of the respective standards. As regards secoiridoids, they have been quantified using the oleuropein standard, due to the lack of the commercial standard. Mass experiments have been done with a Q Exactive Orbitrap LC-MS/MS (Thermo Fisher Scientific, Waltham, MA, USA) equipped with an ESI source (HESI II, Thermo Fisher Scientific, Waltham, MA, USA) operating in the negative ion mode (ESI-). The ion source parameters were spray voltage −3.0 kV, sheath gas (N_2_ > 95%) 30, auxiliary gas (N_2_ > 95%) 15, capillary temperature 200 °C, S-lens RF level 50 and auxiliary gas heater temperature 305 °C. The detection of the MS (Mass) was carried out in two acquisition modes, one in full scan (negative-ion modes) and the other in targeted selected ion monitoring. The parameters of the full scan acquisition mode were: mass resolving power 35,000 full width at half maximum (at *m*/*z* 200), scan range 100–1500 *m*/*z*, scan rate 2s^−1^, and the automatic gain control target 1 × 105 ions for a maximum injection time of 200 ms. The parameters of the targeted selected ion monitoring acquisition mode were: 15 s-time window, resolution power 35,000 full width at half maximum (at *m*/*z* 200), and quadrupole isolation window 1.2 *m*/*z*. The mass list containing exact masses and expected retention times of the target polyphenolic compounds are reported in supplementary material (Table S1).

### 4.7. Aromatic Profile 

Volatile organic compounds (VOCs) were extracted and analyzed from extra virgin olive oil following the method proposed by [18,45] with modifications. Briefly, 2 g of sample were weighed into a 10 mL vial, which was placed in a thermal bath at a temperature of 40 °C for 30 min. Volatile compounds were extracted from the headspace by solid-phase microextraction (SPME) using a 2 cm long Stable Flex (Supelco) fiber, coated with a 30/50 μm layer of divinylbenzene/carboxen/polydimethylsiloxane (DVB/CAR/PDMS).

Subsequently, the SPME fiber was introduced directly into the GC injector, where the thermal desorption of the analytes was performed at 250 °C for 10 min. A 6890N GC system equipped with a 5973 mass detector was used. VOCs were separated on a 30 m × 0.250 mm capillary column coated with a 0.25 μm polymer of 5% diphenyl 95% dimethylpolysiloxane. Splitless injection was used for the samples.

The oven temperature was held at 40 °C for 5 min and increased from 40 °C to 80 °C at 5 °C/min, from 80 to 200 °C at 10 °C/min and from 200 to 270 to 10 °C/min, the temperature of 270 °C was maintained for 4 min. The injection source and ion temperatures were 250 and 230 °C, respectively. Helium was used as a carrier gas at a flow rate of 1 mL/min. The ionizing electron energy was set to 70 eV and the scanned mass range was set to 40–450 amu in the full scan acquisition mode.

Compounds were identified comparing the mass spectra fragmentation patterns with the spectra data from the NIST Atomic Spectra Database version 1.6 and the retention indices with those reported in literature. The relative content of VOCs was calculated on the basis of peak area ratios. 

### 4.8. Statistical Analysis

All the analyses were performed in triplicate. All the analyzed parameters are presented as mean ± standard deviation and calculated by using XLStat version 2009.3.02 software (Addinsoft, Paris, France).

## 5. Conclusions

The results obtained from the study about cv “Oliva Bianca” highlighted interesting qualitative and quantitative aspects; in particular, the drupes showed physical parameters, such as the weight and flesh/pit ratio, suitable also for use as table olives, also showing a high content of anthocyanins, compounds important for their antioxidant effect. Additionally, “Oliva Bianca” oil has shown positive characteristics on human health, such as a high content of oleic acid, in fact recent findings revealed that oleic acid, the main olive oil’s monounsaturated fatty acid, plays a key role in the prevention of various human cancers [50].

Oil extracted has all the characteristics to be considered extra virgin olive oil and the estimated aromatic profile is characterized by a high content of trans-2-hexenal that influences the taste of green, pungent, bitter fruity, apple, almond and cut grass. The high level of oleocanthal attributes to the oil interesting nutraceutical properties considering its considerable positive biological effects. 

Oliva Bianca is only one of “minor” indigenous cultivars of Campania that due to the region’s pedo-climatic conditions and to its large varietal heritage are able to produce oils with high typicity, each significantly different from the other. Our trial is only a starting point for the local olive trees varietal characterization, which allows one to enhance little known cultivars. Nevertheless, additional studies during next years are required to better characterize olive genetic resources in Campania.

## Figures and Tables

**Table 1:** Physical, chemical and chromatic parameters at harvest of the drupes of cultivar “Oliva Bianca”. The morphological parameters of fruit are reported as means ± SD of 100 different replicates (*n* = 100), while the analytical parameters are reported as means ± SD of three different replicates (*n* = 3).

Chemical-Physical Parameters Values	
Drupe weight (g)	4.31 ± 0.90
Flesh weight (g)	3.36 ± 0.80
Pit weight (g)	0.95 ± 0.23
Flesh/pit ratio (Fw/Fw)	3.68 ± 1.00
Polar diameter (mm)	21.41 ± 1.75
Transversal diameter (mm)	17.53 ± 1.29
Pulp firmness (kg/cm^2^)	460.45 ± 84.98
Pigmentation Index (PI)	2.41 ± 0.35
L*	46.45 ± 12.2
a*	6.34 ± 9.62
b*	16.9 ± 15.56
IC	−32.86 ± 1072.93
Anthocyanins (mg/kg)	116.10 ± 0.4
Carotenoids (mg/kg)	2.10 ± 0.01
Chlorophyll a	3.60 ± 0.03
Chlorophyll b	2.10 ± 0.01
Total chlorophyll	5.70 ± 0.01
Water content (%)	62.97 ± 0.17
Oil content (% FW)	18.63 ± 0.28

**Table 2:** Quality indices and panel test of extra virgin olive oil extracted by cultivar “Oliva Bianca”. The parameters are reported as means ± SD of three different replicates (*n* = 3).

Oil Quality Index Values			
		Free acidity (% oleic acid per 100 g oil)	0.51 ± 0,04
		Number of peroxides (meq O_2_ per kg oil)	4.92 ± 0.49
		K_270_	0.09 ± 0.01
		K_232_	1.64 ± 0.31
		Delta K	<0.01
**Panel test**		ATTRIBUTES	SCORE
	NEGATIVE	Heating/sludge	0
		Mold/moisture/ground	0
		Winey/acidic/acid/sour	0
		Frozen olives	0
		Rancid	0
	POSITIVE	Fruity	5.2
		Bitter	3.3
		Spicy	4.7

**Table 3:** Phenolic compounds profile of olive oil of cultivar “Oliva Bianca”. The parameters are expressed as media of three different replicates ± SD (LOD = 2ppb).

Compounds	mg/kg
Hydroxytyrosol	11.98 ± 0.57
Tyrosol	16.45 ± 0.05
Vanillic acid	0.51 ± 0.03
p-coumaric acid	1.09 ± 0.02
Ferulic acid	0.07 ± 0.001
Luteolin rutinoside	0.11 ± 0.01
Elenolic acid	66.70 ± 1.03
Verbascoside	0.002 ± 0.0001
Oleuropein	<LOD
DHPEA-EDA	33.43 ± 0.05
Ligstroside	0.003 ± 0.0002
p-HPEA-EDA	255.20 ± 7.15
Hydroxy Oleuropein aglycon	2.35 ± 0.11
Luteolin	17.25 ± 2.43
3,4-DHPEA-AC	35.45 ± 1.81
DHPEA-EA	85.93 ± 0.47
p-HPEA-EA	122.43 ± 3.86
Total polyphenols	648.95 ± 2.8

**Table 4:** Fatty acid composition of olive oil of cultivar “Oliva Bianca”. The parameters are reported as means ± SD of three different replicates (*n* = 3).

Fatty Acids		%
Palmitic	C16	12.87 ± 0.21
Palmitoleic	C16:1	0.85 ± 0.03
Heptadecanoic	C17	0.06 ± 0.00
Stearic	C18	2.32 ± 0.03
Oleic	C18.1n9c	74.82 ± 0.34
Linoleic	C18:2 Z 9, 12	7.88 ± 0.11
Arachidic	C20	0.37 ± 0.02
Linolenic	C18:3n3	0.74 ± 0.00
Cis-11,14-eicosadienoic	C20:2	nd
Behenic	C22	0.09 ± 0.01
Monounsaturated fatty acids (MUFA)		75.68 ± 0.31
Polyunsaturated fatty acids (PUFA)		8.63 ± 0.11
Saturated fatty acids (SFA)		15.70 ± 0.19
Oleic/linoleic		9.49 ± 0.17
MUFA/PUFA		8.77 ± 0.15
MUFA/SFA		4.82 ± 0.08

**Table 5:** Volatile organic compounds of olive oil of cultivar “Oliva Bianca” oil. The parameters are reported as means ± SD of three different replicates (*n* = 3).

Compounds	% COV
Ethanol	2.93 ± 0.47
Ethyl acetate	3.60 ± 0.37
1-penten-3-one	2.52 ± 0.09
trans-2-hexenal	72.30 ± 1.12
trans-2-hexen-1-ol	7.75 ± 0.71
Hexanol	3.34 ± 0.45
(t, t) 2,4 hexadienal	0.61 ± 0.10
3-ethyl-1,5-octadiene	1.83 ± 0.14
4,8-dimethyl-1,7-nonadiene	0.58 ± 0.07
cis-3-Hexenyl acetate	0.26 ± 0.02
β-ocimene	1.59 ± 0.13
Methyl benzoate	1.11 ± 0.17
Nonanal	0.56 ± 0.01
1,7-octadien-3-one, 2 methyl-6-methylene	0.69 ± 0.11
α-cubebene	0.34 ± 0.02

## Data Availability

The datasets generated for this study are available on request to the corresponding author.

## Supplementary Materials

The following are available online at https://www.mdpi.com/article/10.3390/plants10061119/s1, Table S1: Summary of peaks and mass spectrometric information obtained by (HRMS-Orbitrap).
