# Supplementary material for: Drupe Characters, Fatty Acids, Polyphenolic and Aromatic Profile of Olive Oil Obtained from “Oliva Bianca”, Minor Autochthonous Cultivar of Campania
Source: Plants (Basel). 2021 May 31;10(6):1119. doi: 10.3390/plants10061119 (PMC8226733; doi:10.3390/plants10061119)
Supplement: Supplementary file 1 [file plants-10-01119-s001.zip › plants-1238381-supplementary.pdf]

Table S1. Summary of peaks and mass spectrometric information obtained by (HRMS-Orbitrap).

		Theoretical mass	Experimental mass	Error
Compounds	Formula	(m/z)		∆ ppm
Hydroxy Oleuropein aglycon	$C_{19}H_{22}O_9$	393,11911	393,11948	0,941
p, HPEA-EDA (Deacetoxy ligstroside aglycone)	$C_{17}H_{20}O_5$	303,1238	303,12415	1,155
Elenolic acid	$C_{11}H_{14}O_6$	241,07176	241,07169	-0,290
Ligstroside	$C_{25}H_{32}O_{12}$	523,1821	523,18079	-2,504
DHPEA-EA (Oleuropein aglycone)	$C_{19}H_{22}O_8$	377,12419	377,12442	0,610
p-HPEA-EA	$C_{19}H_{22}O_7$	361,12928	361,12961	0,914
3,4-DHPEA-AC	$C_{10}H_{12}O_4$	195,06628	195,0659	-1,948
verbascoside	$C_{29}H_{36}O_{15}$	623,19814	623,19952	2,214
oleuropein	$C_{25}H_{32}O_{13}$	539,17701	539,17792	1,688
DHPEA-EDA	$C_{17}H_{20}O_6$	319,1187	319,11902	1,003
tyrosol	$C_{10}H_{12}O_4$	137,0608	137,05983	-7,077
OH-tyrosol	$C_8H_{10}O_3$	153,05572	153,05484	-5,750
Pinoresinol	$C_{20}H_{22}O_6$	357,13436	357,1337	-1,848
Acetoxypinoresinol	$C_{22}H_{24}O_8$	303,17544	303,17511	-1,088
Vanillic acid	$C_8H_8O_4$	167,03498	167,03426	-4,310
Ferulic acid	$C_{10}H_{10}O_4$	193,05063	193,04971	-4,766
Coumaric acid	$C_9H_8O_3$	163,03917	163,03931	0,859
luteolin rutinoside	$C_{27}H_{30}O_{15}$	593,15119	593,15222	1,736
luteolin	$C_{15}H_{10}O_6$	285,04062	285,04083	0,737
